# Temporal Trends (1999–2015) in the Impact Factor of Biomedical Journals Published by US and EU Scientific Societies

**DOI:** 10.5041/RMMJ.10332

**Published:** 2018-04-19

**Authors:** Matthew E. Falagas, Margarita Kyriakidou, George Spais, Efstathia Argiti, Konstantinos Z. Vardakas

**Affiliations:** 1 Alfa Institute of Biomedical Sciences (AIBS), Athens, Greece; 2 Department of Medicine, Henry Dunant Hospital Center, Athens, Greece; 3 Department of Medicine, Tufts University School of Medicine, Boston, MA, USA; 4 Department of Applied Mathematical and Physical Sciences, National Technical University of Athens, Athens, Greece

**Keywords:** Impact factor, EU, USA

## Abstract

**Objective:**

The impact factor has emerged as the most popular index of scientific journals’ resonance. In this study we aimed to examine the impact factor trends of journals published by scientific bodies in the United States of America (USA) and Europe (EU).

**Methods:**

We randomly chose 11 categories of Journal of Citation Reports and created three research classes: clinical medicine, laboratory medicine, and basic science. The impact factor values for the years 1999–2015 were abstracted, and the impact factor of US and EU journals was studied through the years.

**Results:**

A total of 265 journals were included in the final analysis. The impact factor of US journals was higher than that of EU journals throughout the study period. In addition, for both US and EU journals the median impact factor increased throughout the study period. The rate of annual change in the impact factor throughout the study period was lower for US than EU journals (1.85% versus 3.55%, *P*=0.019). A higher median annual increase was seen in the impact factor during the period 1999–2008 compared to the period 2009–2015 for both US (*P*<0.001) and EU (*P*=0.001) journals. In fact, during the second period the US median impact factor value did not show significant changes (*P*=0.31), while the EU median impact factor continued to increase (*P*<0.001).

**Conclusion:**

The impact factor of EU journals increased at a significantly higher rate than and approached that of the US journals during the last 16 years.

## INTRODUCTION

Articles published in scientific journals are the means through which advances in science and innovative, breakthrough research ideas from reputable scientists are communicated to peers throughout all scientific fields. As the number of journals increased,^1^ several indices were developed to measure the “quality” or “importance” of a journal.^2^,^3^ Despite its limitations and in some cases misuse,^4^–^6^ the impact factor has emerged as the most popular index of each journal’s influence in the scientific community.

In a previous analysis, we studied the variations of impact factors of European and American journals, in selected years during a 10-year period (1999, 2002, 2005, and 2008).^7^ That study focused on four categories (biology, cell biology, critical care medicine, and infectious diseases) and showed that European journals’ impact factors in some cases increased by a higher percentage than those of American journals. In this study, we aimed to examine the impact factor trends in a more general aspect and to investigate if the gap between impact factor of journals published by scientific bodies in the United States of America (US) and Europe (EU) continues to decrease in three major scientific fields: clinical medicine, laboratory medicine, and basic science.

## METHODS

The Incites Journal of Citation Reports of the Web of Science classifies scientific journals into 22 scientific research fields which are further divided into 227 categories.^8^ From these categories we excluded those that do not have direct or indirect impact on medicine. From the remaining categories we randomly chose 11, to create three research classes: clinical medicine, laboratory medicine, and basic science. Randomization was performed by assigning a number to each of the 227 categories (following classification to the three classes) and randomly selecting from them using the random number function of random.org. Acknowledging that the orientation of a journal may not be straightforward and that several of them may publish articles from >1 of the selected research classes, the classification of the Web of Science categories was performed according to the “highest possible” share of published articles in clinical and laboratory medicine and basic science.

Data regarding impact factors derived from the Web of Science, Incites Journal Citation reports.^8^ Only journals published on behalf of scientific societies or institutions (universities, foundations, etc.) were eligible for inclusion. The study period was divided into two sub-periods, 1999–2008 and 2009–2015, to account for the possible impact of the economic crisis that started in 2008. The impact factor values for the years 1999–2015 were abstracted, and the impact factor variation was studied through the years. For the analysis we used journals that were included in each of the randomly selected categories at the year 1999. If the impact factor for more than 75% (12/16) of the years 1999–2015 for a given journal was not available (due to discontinuation in production, change of its name, removal from the specific category according to Web of Science criteria, or non-specified etiology), the journal was not included in the analysis.

We used Excel files to create the databases, one for journals of each class and one with the total number of studied journals. The normality of the distribution of the impact factors in each year was tested by the Kolmogorov–Smirnov test. If the distribution was normal we used the mean of the values; if not, we used median. The distribution for the most part was expected to be non-normal, therefore to test if the median impact factor of US or EU fluctuates or remains the same throughout the years we used the Friedman test. The difference between the two periods (1999–2008, 2009–2015) was studied with Wilcoxon–Mann–Whitney test. For all comparisons, statistical significant differences were denoted if the *P* value of the test was ≤0.05. Statistical analyses were performed using SPSS (SPSS v.23)^9^ and Microsoft Excel (Microsoft, 2007) computer software.

## RESULTS

The randomly selected categories were: for clinical medicine, “emergency medicine,” “endocrinology–metabolism,” and “hematology and oncology”; for laboratory medicine, “genetics heredity,” “immunology,” “pathology,” and “radiology, nuclear medicine, and medical imaging”; and for basic science, “statistical probability,” “chemistry medicinal,” and “physics, atomic, molecular, and chemical.” The 11 categories included in the study contained a total number of 874 journals. After the exclusion of 609 journals (395 were not published on behalf of a scientific society; 154 could not be categorized in either the EU or US group; 5 were included in the Web of Science categories more than once; and 55 for which data for less than 75% of the study period was available), 265 journals were included in the final analysis; 107 of the initial 273 (39.2%) regarding clinical medicine, 103 out of 348 (29.6%) of laboratory medicine; and 55 out of 253 (21.7%) of basic science.

**Figure 1:**
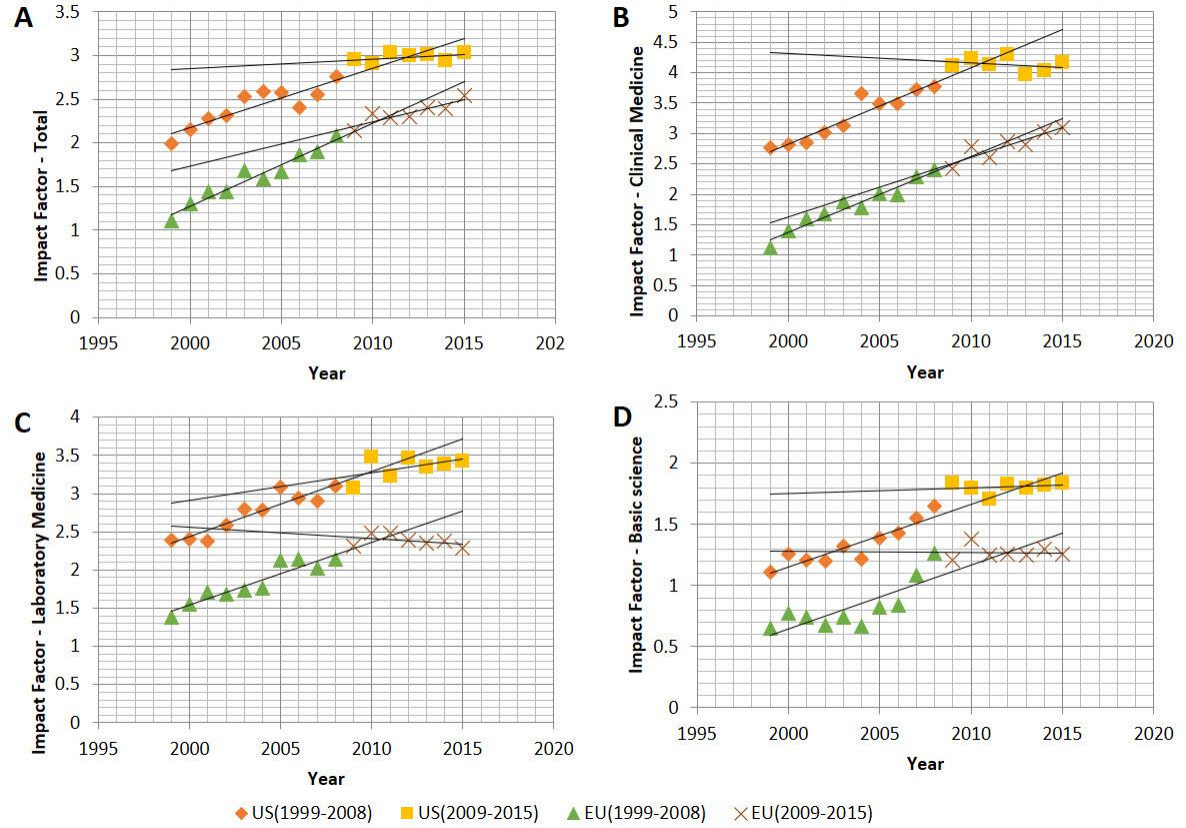
Impact Factor Trends of US and EU Journals during the Study Period (1999–2015) **A:** All journals. **B:** Clinical medicine journals. **C:** Laboratory medicine journals. **D:** Basic science journals.

Figure 1A shows that the impact factor of US journals was higher than that of EU journals throughout the study period. In addition, for both US and EU journals the median impact factor increased throughout the study period. The same was true for all three classes (clinical medicine, laboratory medicine, and basic sciences). The rate of annual change in the impact factor throughout the study period was lower for US than EU journals (1.85% versus 3.55%, *P*=0.019). However, the difference in the annual percentage increase of impact factor between US and EU journals was not significant for the two sub-periods 1999–2008 (3.30% versus 4.80%, *P*=0.08) and 2009–2015 (0.59% versus 2.46%, *P*=0.07). A higher median annual increase was seen in the impact factor during the first period compared to the second for both US (*P*<0.001) and EU (*P*=0.001) journals. In fact, during the second period the US median impact factor value did not show significant change (*P*=0.31), while the EU median impact factor continued to increase (*P*<0.001).

Figure 1B–D presents the median impact factor for the three classes per year. The fluctuation of median impact factor was different for the three classes. In general, an increase during the period 1999–2008 was observed for all three classes and for both US and EU journals. The pattern was distinctly different during the period 2009–2015. In clinical medicine, the median US impact factor tended to decrease, while the EU impact factor continued to increase, although at a lower rate than that of the first period. In laboratory medicine, the reverse phenomenon was observed as the median US impact factor continued to increase (although at a lower rate than that of the first period), while the EU impact factor tended to decrease. Finally, both US and EU median impact factors in basic science tended to be stable during the second period.

## DISCUSSION

The main findings of this study were the convergence of the US and the EU median impact factor values and the trends in the course of the median impact factor throughout the years, especially after the year 2008. European journals’ impact factor values tended to approach the US median value during the study period. Although the impact factor of both US and EU journals increased during the first period, the US median impact factor remained almost unchanged throughout the years 2009–2015, while EU median continued to increase. This should probably be attributed to the continued increase of the impact factor of journals in clinical medicine.

Several causes may have led to the convergence of US and EU journal impact factors. The funds made available for biomedical research by each region could be a plausible explanation. Recent reports described a decline of available funds by approximately $12 billion from 2007 to 2012 (cumulatively $49 billion less compared to a constant 2007 spending) in the US, leading to a decline in the US share of global biomedical research and development from 51.2% in 2007 to 45.4% in 2012.^10^ The decrease was due to a decline in industry investment, since the public-sector contribution was stable. The corresponding value for the EU decreased by $1.8 billion (but cumulatively $5.2 billion more compared to a constant 2007 spending), resulting in a minor increase in the EU share from 28.5% to 29.2%.^10^ In addition, there has been a reduction of funding by the National Institutes of Health following 2013 and increased spending and better organization of the allocated resources in the EU.^7^

Available funds may affect not only the number of performed studies but also their quality. The impact factor could be affected by both. Scientists (and journals) tend to cite their own studies or studies from the journals they publish in.^11^–^15^ Thus, the increase in the number of articles from EU-based scientists published in EU-based journals could have favored the increase in EU journal median impact factor.^1^ Furthermore, local European journals tended to change the publication language from the national language to English in order to increase readership and potentially impact, i.e. the impact factor.^7^,^16^,^17^ The visibility of EU journals could have increased by the introduction of other databases like Scopus that include more EU journals than PubMed or Web of Science.^6^ In addition, a possible factor in the dissemination of articles might have been the online version of articles.^18^,^19^ Finally, “cross-publishing” (publication of EU studies in US journals and vice versa) can be another reason, as authors have the chance to decide in which journal to publish their study. A journal with higher impact factor could be a more attractive choice for article submission.^5^ Thus, US authors may choose to publish potentially high-impact articles in EU journals with higher impact factor than US journals.

The findings of the study are limited by the incorporation of journals from only 11 Web of Science categories. In addition, the findings probably do not apply to all scientific fields, as only categories closely related to medicine were included in the analysis. Another limitation could be the classification of journals in Web of Science fields as clinically, laboratory-, or basic science-oriented. Several journals may publish articles from all three classes, precluding an exact classification. In addition, the same journal can be included in different Web of Science categories. A more exact classification would have required access to the individual papers, but this would not serve the aim of this study. Furthermore, the effect of open-access publication type on the impact factor trends among US and European journals was not evaluated in the current study. It has been shown that open-access articles are cited at a higher rate compared to those requiring subscription.^20^,^21^

It could also be argued that the small sample of journal categories may not be representative of the entire biomedical field. However, the categories were randomly selected, which decreases the degree of bias. Furthermore, we observed differences between the selected classes, in accordance with other studies that evaluated similar outcomes.^22^ The study is also limited by the inclusion of journals issued by scientific societies only, listed in the specific category in 1999, and for which data were available for the great majority of the study period. Although these decisions decreased the available number of journals, in our opinion it provided the best possible means for inclusion of journals with accurate geographic origin (mainly the scientific board) and complete data for the entire study period.

In conclusion, despite the higher impact factor of US journals throughout the study period, in total and in the three individual classes, the impact factor of EU journals increased at a significantly higher rate than and approached that of the US ones during the last 16 years. Distinct differences in the fluctuation of impact factors were seen between journals in clinical and laboratory medicine as well as basic sciences.
